# Evaluating Cognitive Enrichment for Zoo-Housed Gorillas Using Facial Recognition

**DOI:** 10.3389/fvets.2022.886720

**Published:** 2022-05-18

**Authors:** Otto Brookes, Stuart Gray, Peter Bennett, Katy V. Burgess, Fay E. Clark, Elisabeth Roberts, Tilo Burghardt

**Affiliations:** ^1^Department of Computer Science, Faculty of Engineering, University of Bristol, Bristol, United Kingdom; ^2^Centre for Entrepreneurship, Faculty of Engineering, University of Bristol, Bristol, United Kingdom; ^3^School of Psychological Science, Faculty of Life Sciences, University of Bristol, Bristol, United Kingdom; ^4^School of Life Sciences, Faculty of Science and Engineering, Anglia Ruskin University, Cambridge, United Kingdom; ^5^Bristol Vet School, Faculty of Life Sciences, University of Bristol, Bristol, United Kingdom

**Keywords:** facial recognition, gorillas, animal welfare, machine learning, zoology, cognitive enrichment

## Abstract

The use of computer technology within zoos is becoming increasingly popular to help achieve high animal welfare standards. However, despite its various positive applications to wildlife in recent years, there has been little uptake of machine learning in zoo animal care. In this paper, we describe how a facial recognition system, developed using machine learning, was embedded within a cognitive enrichment device (a vertical, modular finger maze) for a troop of seven Western lowland gorillas (*Gorilla gorilla gorilla*) at Bristol Zoo Gardens, UK. We explored whether machine learning could automatically identify individual gorillas through facial recognition, and automate the collection of device-use data including the order, frequency and duration of use by the troop. Concurrent traditional video recording and behavioral coding by eye was undertaken for comparison. The facial recognition system was very effective at identifying individual gorillas (97% mean average precision) and could automate specific downstream tasks (for example, duration of engagement). However, its development was a heavy investment, requiring specialized hardware and interdisciplinary expertise. Therefore, we suggest a system like this is only appropriate for long-term projects. Additionally, researcher input was still required to visually identify which maze modules were being used by gorillas and how. This highlights the need for additional technology, such as infrared sensors, to fully automate cognitive enrichment evaluation. To end, we describe a future system that combines machine learning and sensor technology which could automate the collection of data in real-time for use by researchers and animal care staff.

## 1. Introduction

Animal technologies in zoos have a long history; they reflect society's changing perceptions of animal intelligence and welfare, and technological advancements and fashions. The use of technology in zoos can be traced back to the research of Hal Markowitz beginning in the late 1970s and the birth of “behavioral engineering” (1, 2). However, the concept of zoo animals interacting with technology for the purposes of enrichment has experienced a renaissance over the past two decades (3–5). Contemporary animal-computer interaction (ACI) usually involves animals directly interacting with components such as touchscreens (4, 6), buttons or joysticks (7, 8), and therefore animals need to be trained to use the technology. Animals may also directly experience technological feedback systems in the form of lights, tones or vibrations (9, 10).

Cognitive enrichment is a form of enrichment that aims to challenge the evolved cognitive skills of animals to enhance their welfare, yet it remains under-provisioned in many zoo settings (11). Previous research has identified cognitive enrichment as being particularly applicable to great apes under human care following decades of study into their cognitive abilities as compared to humans, and their swift adaptation to novel phenomena (12). We believe there is great potential to embed technology within cognitive enrichment and there are several possible avenues for realizing this. Digital interfaces can augment how cognitive challenges are provided to animals in their enclosure (e.g., the contrast between a tangible and digital maze, for example), and furthermore, provide more novel and repeatable experiences across time (13). Technology also creates opportunities for instant feedback and to automate processes such as food reward presentation (13–15). For the focus of this paper, however, we are interested in the use of technology to automatically log an animal's response to a cognitive enrichment device, foremost to save researcher time and effort, but also to prevent human observation from influencing animal behavior. Cognitive enrichment requires us to simultaneously measure how the animal is performing cognitively (i.e., their learning, memory, problem-solving skills), and their welfare state (i.e., if their wellbeing is positively affected by the enrichment). In other words, we must evaluate the cognitive challenges presented by enrichment phenomena and to know whether it is eliciting the intended cognitive skills, as well as any emotional or behavioral affect it is having upon the animals involved (16). Cognitive enrichment devices may therefore generate relatively “big data” that zoo-based researchers and animal care staff are not accustomed to collecting. It thus follows that if some, or all, of this data collection could be automated, we can maximize our understanding of the cognitive and behavioral implications of such enrichment devices.

Full, empirical enrichment evaluation requires data such as (i) which individual/s are present, (ii) their duration and frequency of enrichment use, and (iii) how the enrichment is being used. These observations are particularly important for cognitive enrichment where enrichment use may not correlate with reward depletion (e.g., an animal may spend a long period of time using enrichment without successfully extracting many rewards). Researchers often have limited time available to observe animals, which in turn can limit the time enrichment is provided to animals and/or scientifically evaluated. The collection of these data, however, is time-consuming as it is typically undertaken live (“online”) by researchers. The collection of these data can be automated using radio frequency identification (RFID) tags. RFID tags are worn on a collar or implanted into the skin and have been used to monitor several laboratory primate species, such as rhesus macaques (*Macaca mulatta*) (17); common marmosets (*Callithrix jacchus*) (18); guinea baboons (*Papio papio*) (19, 20). However, there are several reasons why this approach may be deemed unsuitable. Primarily, it classifies as invasive research and may therefore be in conflict with the researchers' code of ethics. Additionally, in our opinion, RFID tags pose a risk to the safety of great apes and implants are notoriously difficult to maintain in primates due to overgrooming (FE Clark, personal communication).

Machine learning offers the potential to automate collection of empirical data using non-invasive techniques. In recent years, machine learning has been widely applied within the field of animal biometrics and several machine learning systems have been developed specifically for great apes. These systems address a wide range of tasks including; individual detection (21), pose estimation (22), and behavior recognition (23). However, in the study presented in this paper, we focus on individual identification. Facial recognition technology for humans has long been prominent within machine learning and computer vision (24, 25). In particular, deep convolutional neural networks (CNNs) (26), exemplified in frameworks such as DeepFace (27), form the basis of most modern facial biometric frameworks (28). Great apes share similar facial characteristics with humans because of our close evolutionary lineage (29). Thus, a number of methodologies in animal biometrics (30) follow approaches for human face recognition closely. Based on these approaches, a number of machine learning systems have been developed for the detection and recognition of great apes in both captive and wild environments (31–33).

In this paper, we extend the development of a new machine learning system for the facial recognition and individual identification of the Western lowland gorillas (34) housed at Bristol Zoo Gardens for specific use with a cognitive enrichment device. This research was undertaken as part of a larger research project called Gorilla Game Lab, a collaborative and interdisciplinary venture between Bristol Zoological Society and the University of Bristol. The project brings together researchers from the fields of animal welfare science, animal psychology, computer vision and machine learning, and human-computer interaction. Together, we evaluate the efficacy of our system and examine and discuss its potential to automate aspects of the traditional evaluative approach, human observation. We report upon the merits of each method, with regards to time and resources, expertise, the value of the resulting data, and to ultimately compare their efficacy. Finally, we speculate about the future use of our facial recognition system within a complementary ecosystem of technologies that would allow for greater automation of enrichment analysis within future zoological environments.

## 2. Methods and Materials

### 2.1. Study Duration and Phases

Data collection took place between May-July 2019. The design and evaluation of the enrichment device employed in this study was published in (13) and (35), respectively. The implementation, training and evaluation details of the machine learning model can be found in (34).

### 2.2. Study Subjects and Housing

Study subjects were a troop of 7 Western lowland gorillas (*Gorilla gorilla gorilla*) housed at Bristol Zoo Gardens (Table 1). Gorillas were housed as one group in the “Gorilla Island” exhibit, comprising a large outdoor island (2,048 m^2^) and an indoor enclosure (161.9 m^2^). Information on gorilla husbandry and feeding is provided in (35).

**Table 1:** Information on gorilla study subjects at Bristol Zoo Gardens.

**Name**	**Sex**	**Age (years)**	**Rearing type**
Jock	M	35	Parent
Kera	F	13	Hand
Touni	F	10	Parent
Kala	F	8	Parent
Kukena	F	7	Parent
Afia	F	2	Hand
Ayana	F	1	Parent

### 2.3. Ethics Statement

Data collection was undertaken with the approval of Bristol Zoological Society and the University of Bristol Animal Welfare and Experimental Research Boards (codes UK/19/021, 84663). Gorillas interaction with the enrichment device was voluntary, and subjects were not food deprived or confined to certain areas of the exhibit during data collection.

### 2.4. Enrichment Device

**Figure 1:**
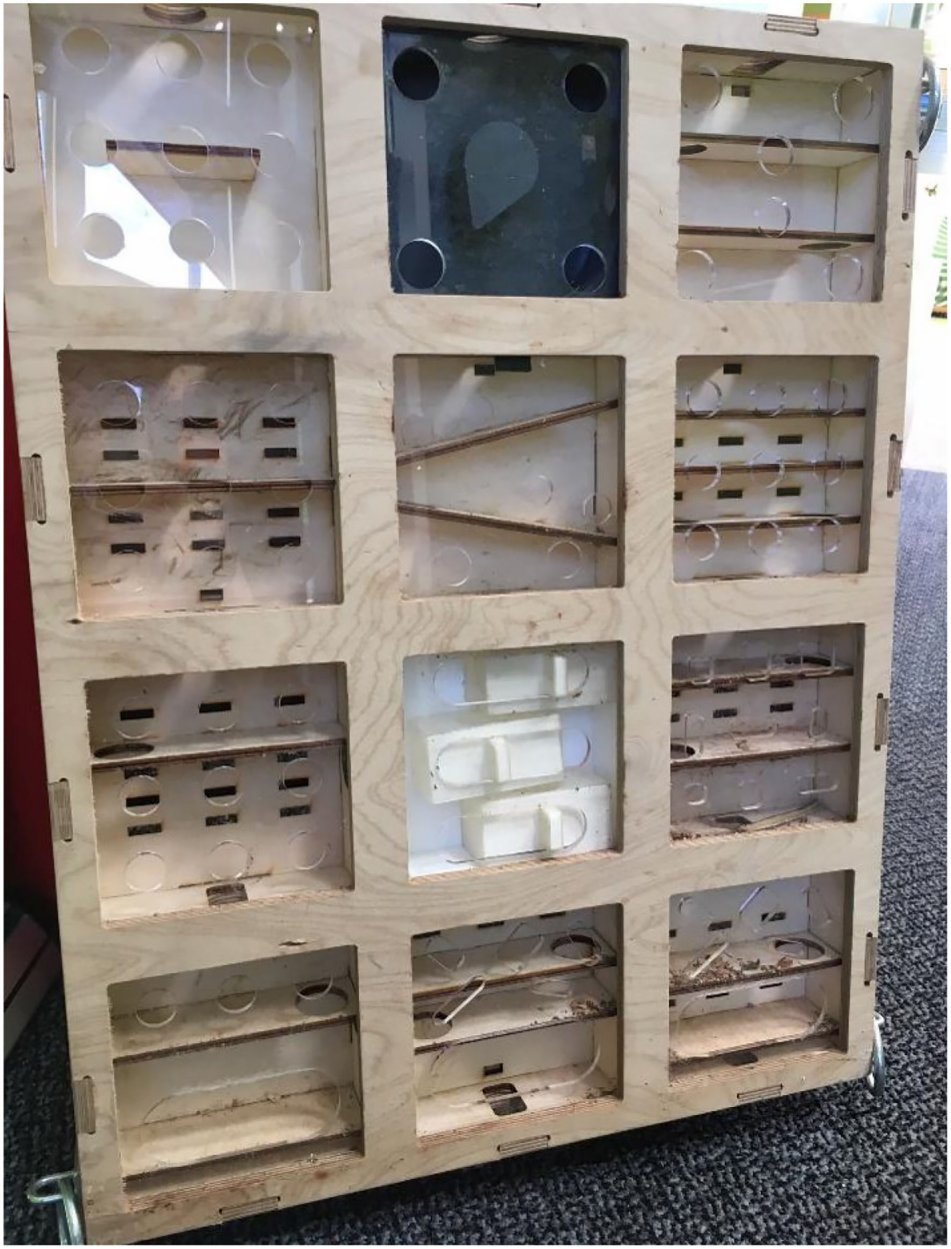
Gorilla Game Lab cognitive enrichment device. A 3D finger maze consisting of 12 puzzle modules. The modular design allows a camera to be fitted inside or on top of the modules. In turn, this allows footage of the gorilla engaging with the device to be recorded.

The Gorilla Game Lab enrichment device is fully described in (35). In summary, it consists of a wooden frame holding 12 removable puzzle modules, in addition to associated video (Figure 1). The device operated independently from the technology; gorillas could use and solve the device without it being connected to a power source or sensors. The technology (to be described in Sections 3.2 and 3.3) was placed behind physical barriers so it could not be tampered with by the gorillas.

The device frame (850 × 650 × 80 mm) and 12 puzzle modules (200 × 200 × 60 mm, arranged in 3 rows and 4 columns) were constructed from plywood, and each module had a front sheet of transparent acrylic with drilled finger holes (30–40 mm) or stick tool holes (15 mm). When placed in the frame, modules were connected to each other *via* 30 mm holes on each side. Components were laser-cut and slotted together with adhesive-free joints so that they could be assembled and re-assembled easily. (13) describes the overall premise of the device design but to summarize, we intended for the gorillas to manoeuvre monkey nuts from the top of the device to the bottom through the interconnected modules. The bottom row of modules had larger openings in the acrylic sheets to allow removal of nuts. The modules varied in design, containing vertical or diagonal ledges, dials, and sliding drawers made from wood and black or white plastic. The device therefore challenged the gorillas' cognitive and motor skills in a number of ways including the stimulation of stick tool use (sticks were provided in the form of clumps of tree branches during data collection).

### 2.5. Data Collection Schedule

The device was presented to gorillas indoors in a ground-level area of the enclosure for 1 h between routine feeding times (11:00–12:00 h). It was attached behind the cage mesh (gauge 100 × 50 mm) with D-shackles so that the front of the modules sat directly behind the mesh. The device was presented 12 times. The same 12 modules used in Phase 1 were presented [see (35)], but in three sets which were swapped between the left, middle, and right columns between trials.

### 2.6. Behavioral Observation

We opted to video record behavioral observations using high-quality cameras positioned at appropriate angles (i.e., non-invasive and discreet but positioned to maximize the view of the gorillas and the device). Although behavioral observations can be captured “live” and by eye, there is only one chance to collect the data and the presence of the researcher may produce an additional environmental effect. Moreover, video recorded footage can be revisited and reviewed by multiple observers, who are able to co-construct a thematic analysis of events rather than relying upon a singular interpretive lens.

#### 2.6.1. Video Camera Footage

Gorilla behavior was captured using two HD cameras with internal batteries. An action camera (GoProHERO 7, GoPro, Inc., CA, United States) was placed on top of the device behind the cage mesh. It faced outwards toward the gorilla/s using it. This camera was switched on during device installation, and left to record footage until the device was uninstalled 1 h later. A second larger camera (Sony HDR-CX405 Handycam Camcorder, Sony Corporation, Tokyo, Japan) was positioned on a tripod in the indoor visitor viewing area, approximately 2.5 m from the device. This camera was manually operated by a researcher who could make adjustments to its location, height and angle during a trial. This was in response to the gorilla and visitor movements and changing natural light levels. It recorded device use “over the shoulder” of the gorilla but at an angle so as much device use could be recorded as possible. Data were stored on SD cards and later downloaded to hard drives. An information sign in the visitor area was used to explain the gorillas were being filmed for a research project.

#### 2.6.2. Video Coding

Video footage was replayed through Windows Media Player^®^ version 10 (Microsoft©, NM, United States). One researcher scored all the footage. All gorillas within an arm's reach of the device were coded from footage from the inward facing camera (Handycam), but on occasion footage from the outward facing camera (GoPro) was needed to confirm if gorillas were looking at the device. The following data were scored using continuous sampling (36, 37) for each gorilla within arm's reach: frequencies and durations of device use (observing/contacting); strategy (observe, types of hand, and mouth use); and module/s used; successes (extraction of food rewards). All data were entered into Microsoft Excel for summary, visualization, and analysis.

### 2.7. Machine Learning

In this study, a deep learning object detection model, YOLOv3 (38), was employed to perform simultaneous facial detection (i.e., localization) and identification of gorillas. That is, YOLOv3 predicts the location of the facial region and the identity of the individual gorilla. As discussed, the system was intended to automate monitoring of device usage (for example, frequency, duration, and order of engagement). Broadly, this was undertaken in two stages; (i) dataset generation and (ii) model implementation and training. Each of these phases is described in the following sections.

#### 2.7.1. Dataset Generation

**Figure 2:**
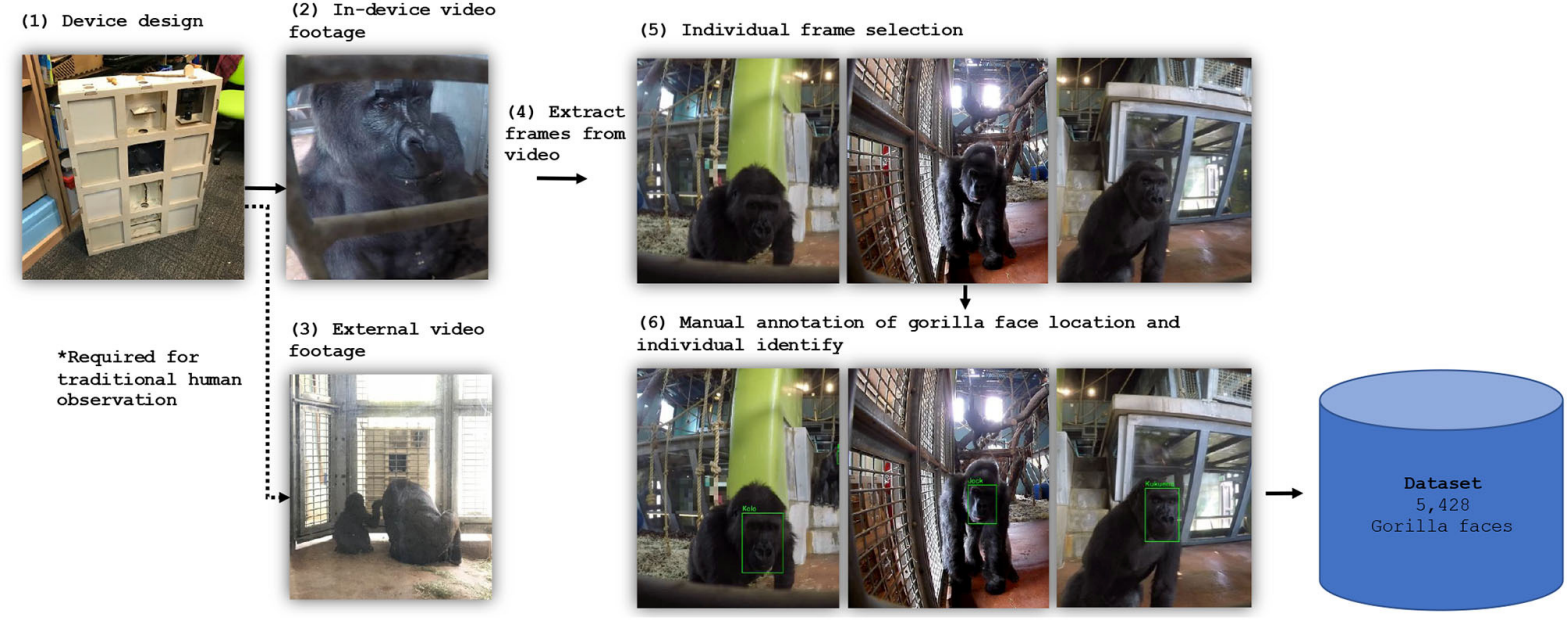
Dataset generation. Cameras recorded front-facial footage of the gorillas (2). Individual frames were extracted from the video footage (4), assessed for suitability and selected for labeling (5). This resulted in 5,248 individual frames being manually annotated with bounding box and identity information (6). Note that (3) forms part of the human observation pipeline.

Machine learning models require data that they can *learn* from. That is, datasets of images or video which are annotated with information of interest (i.e., species, location, identity, etc.) about the animal subjects. Therefore, it was necessary to curate a custom dataset comprising a representative sample of images for each gorilla in the troop (see Figure 2 for an overview of this phase). The footage gathered for the machine learning aspect of the project was not obtained from the camera in the GGL device, as shown in Figure 1, but from the 4 custom-built modules, as described below.

**Data collection**. Four modules were designed to securely hold GoPro (versions 5 & 7) and Crosstour Action cameras that could be safely installed in the gorilla enclosure. The camera modules were distributed throughout the gorilla enclosure. Each of the modules were positioned near the Gorilla Game Lab device and other enrichment objects to obtain close-up footage of the gorillas. Two of the cameras were situated near the Gorilla Game Lab device, allowing representative footage of the gorillas engaging with the device to be captured. To maximize the footage gathered for each individual gorilla and allow for a balanced dataset to be generated each of the cameras recorded footage simultaneously. The zookeepers at Bristol Zoo observed that enrichment devices are dominated by higher-ranking members of the troop. If the higher-ranking members of the troop used the enrichment devices for the duration of the data collection session then no footage of the lower- ranking members would be captured. It was therefore important to devise a strategy to gather enough data for each individual. Data collection sessions took place twice per week from 11:00 to 13:00 h over a period of 6 weeks in study phase 2. During each session, each camera recorded approx. 2 h of footage RGB video at 1,280 × 720 pixels and 30 FPS.

**Data Processing**. The raw footage obtained from the cameras was retrieved as 30-min segments. Video segments were played back and edited into several sub-segments, each containing footage of the dominant gorilla in frame. The background of the footage was blurred using software to remove any humans (visitors, staff) inadvertently captured (although this was rare due to the lighting through the enclosure glass). After each segment was processed, suitable frames containing un-occluded front facial images were selected for labeling with location and identity. As a result of the erratic movements of the gorillas (i.e., rapid changes in poses, movement into spaces occluded by the mesh, tampering with the camera-housing modules, etc.) it was necessary to perform this process manually.

**Figure 3:**
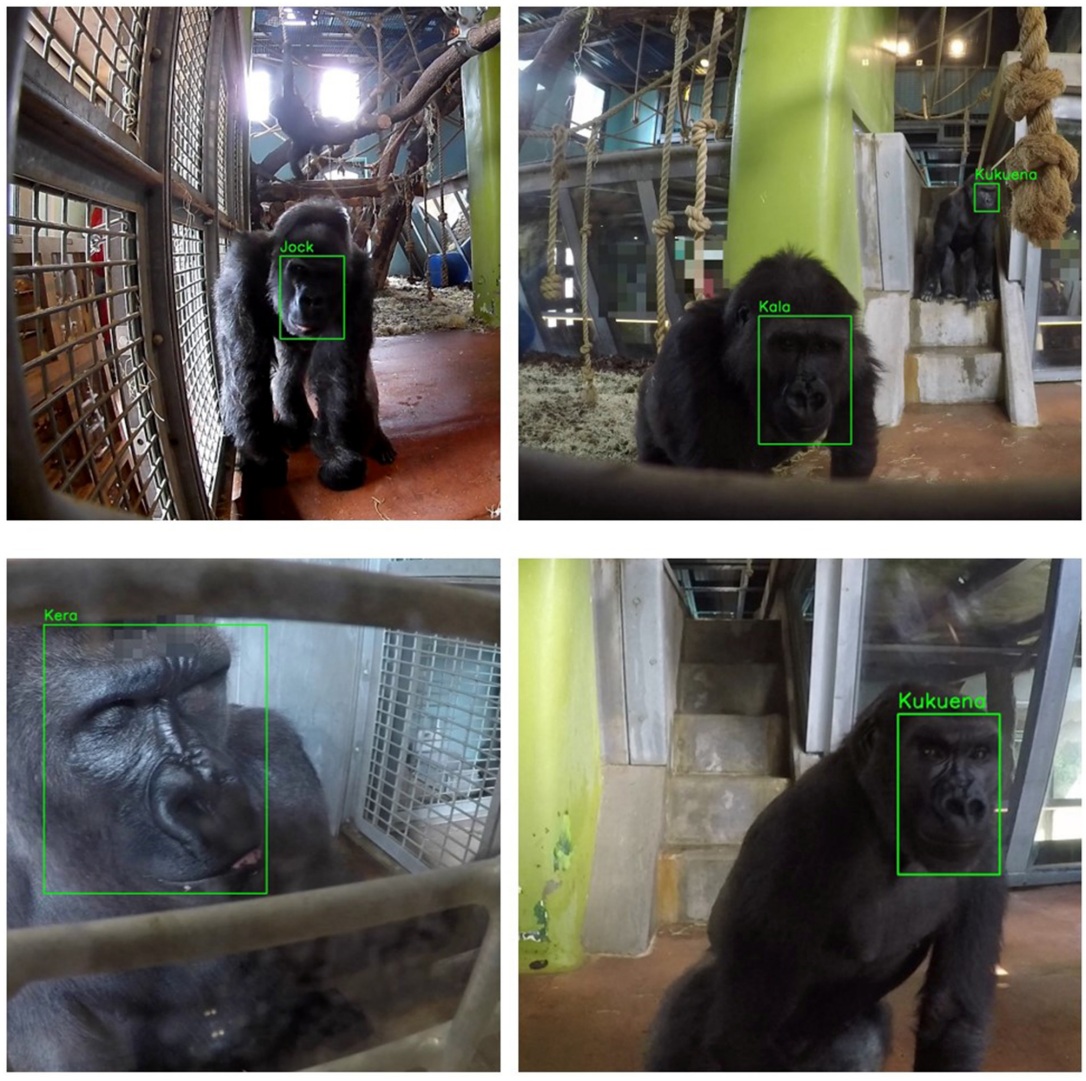
Gorilla face annotation. The figure shows a collection of images that were annotated during the dataset generation process. The green boxes were manually annotated (or drawn) onto the images to indicate the location of the gorillas face. This was done by dragging a rectangle over the facial area from top-left to bottom-right. In these images the name of the gorilla (or *class*) is shown for clarity.

Labeling is essential for supervised machine learning models. It is the process of generating ground truths and is therefore required by the model's learning algorithm. In this project, an image label includes the class, corresponding to each gorilla's name, and a set of coordinates that specify the center (*x, y*), height (*h*) and width (*w*) of the enclosing bounding box relative to the input image size. Image annotation was undertaken manually with the help of primate keepers at Bristol Zoo Gardens using the LabelImg tool.^1^ This ensured the identities of individual gorillas were labeled correctly. Each of the selected images was annotated with the class (i.e., the gorillas identity) and location of the corresponding gorillas face, as illustrated in Figure 3. Only frames that were sufficiently different were selected to ensure diversity.

The complete dataset was further processed to generate training and validation datasets; the training and validation sets are prepared by randomly sampling 80 and 20% of the images gathered for each gorilla, respectively. This was intended to preserve any class imbalances and ensured the validation set was representative of the complete dataset. The dataset was also partitioned into five-folds to allow stratified cross-validation to be performed. This was done by randomly partitioning the entire dataset into five independent folds, each comprising 20% of the dataset. Random sampling is performed for each class to ensure imbalances are preserved between folds. This allows each image to be used in the *k*^*th*^ validation set once and used to train the model *k*−1 times.

#### 2.7.2. Model Implementation

**Figure 4:**
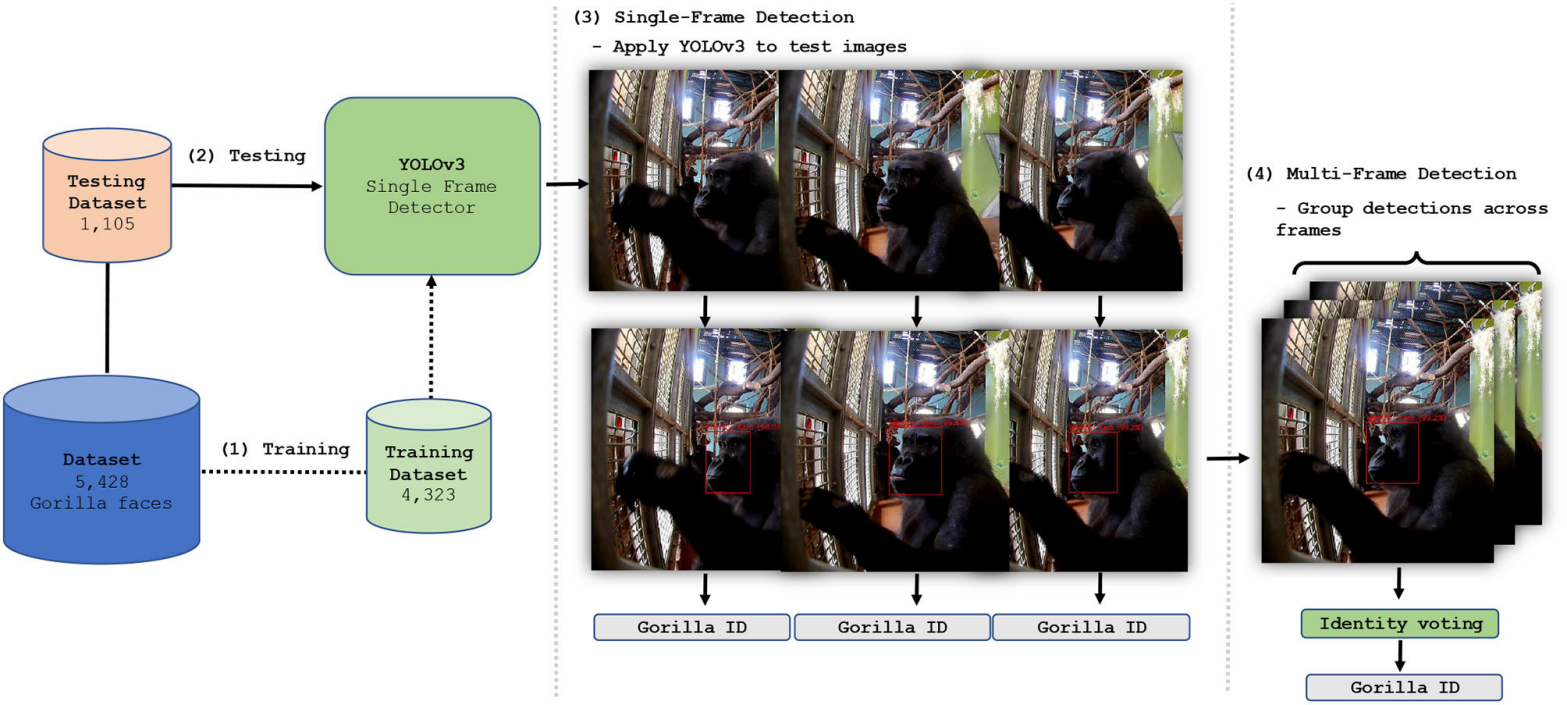
Facial recognition model implementation and training. The dataset was used to train (1) and test (2) a YOLOv3 detection and classification system. The system was tested as a single frame recognition system (3), and a multi-frame system, (4) yielding facial location predictions and gorilla identities.

The model's ultimate objective is to ingest data (i.e., an image of a gorilla) and predict information of interest (i.e., facial region location and identity of the gorilla). The model learns to do this through the process of *training*. During training, the model is exposed to input-target pairs, where inputs correspond to data (i.e., an image of a gorilla) and targets correspond to information of interest (i.e., the corresponding facial location and individual identity). Through training, the model learns to extract features from the inputs which allow them to be mapped to the targets i.e., allowing facial region location and individual identities to be predicted from input images. Therefore, once the dataset (which comprises input-target pairs) had been prepared, as described in Table 2, we began training YOLOv3 on the task of facially recognizing individual gorillas in single frames. Once the standalone performance of YOLOv3 had been optimized, a multi-frame approach which utilizes temporal information to assist with ID's was developed. Details of both single-frame and multi-frame applications are given below (see Figure 4 for an overview of this phase).

**Table 2:** Complete dataset.

**Gorilla name**	**Training**	**Validation**	**Total images**
Afia	614	157	771
Ayana	489	126	615
Jock	387	101	488
Kala	578	148	726
Kera	776	196	972
Kukena	747	190	937
Touni	732	187	919
Total	4,323	1,105	5,428

*The dataset comprises 628 video segments and 5,428 annotated facial images (sampled from the corresponding video segments)*.

To train YOLOv3 the freely available Darknet software (38) was used and several mechanisms known to improve performance were employed. First, we use a model already trained to classify 1,000 different classes on the ImageNet-1000 dataset (39). This is know as *pre-training*. It provides the model with some basic prior knowledge of what is important when classifying images and is task-agnostic. Additionally, the *k*-means algorithm was applied to the training dataset to generate anchor boxes. Anchor boxes are used as a reference (38) from which predictions can be made by the model. By generating them using the training data, the model is provided with task-specific prior knowledge of facial localization information. We also applied several augmentation methods. This included random transformation of the training images using saturation, exposure, hue distortion, cropping and flipping. Furthermore, instead of fixing the input image size the model randomly chooses a new image resolution size every 10 batches. These augmentation methods enable the model to classify apes regardless of variation in images (for example, input dimensions or varied lightning conditions).

**Figure 5:**
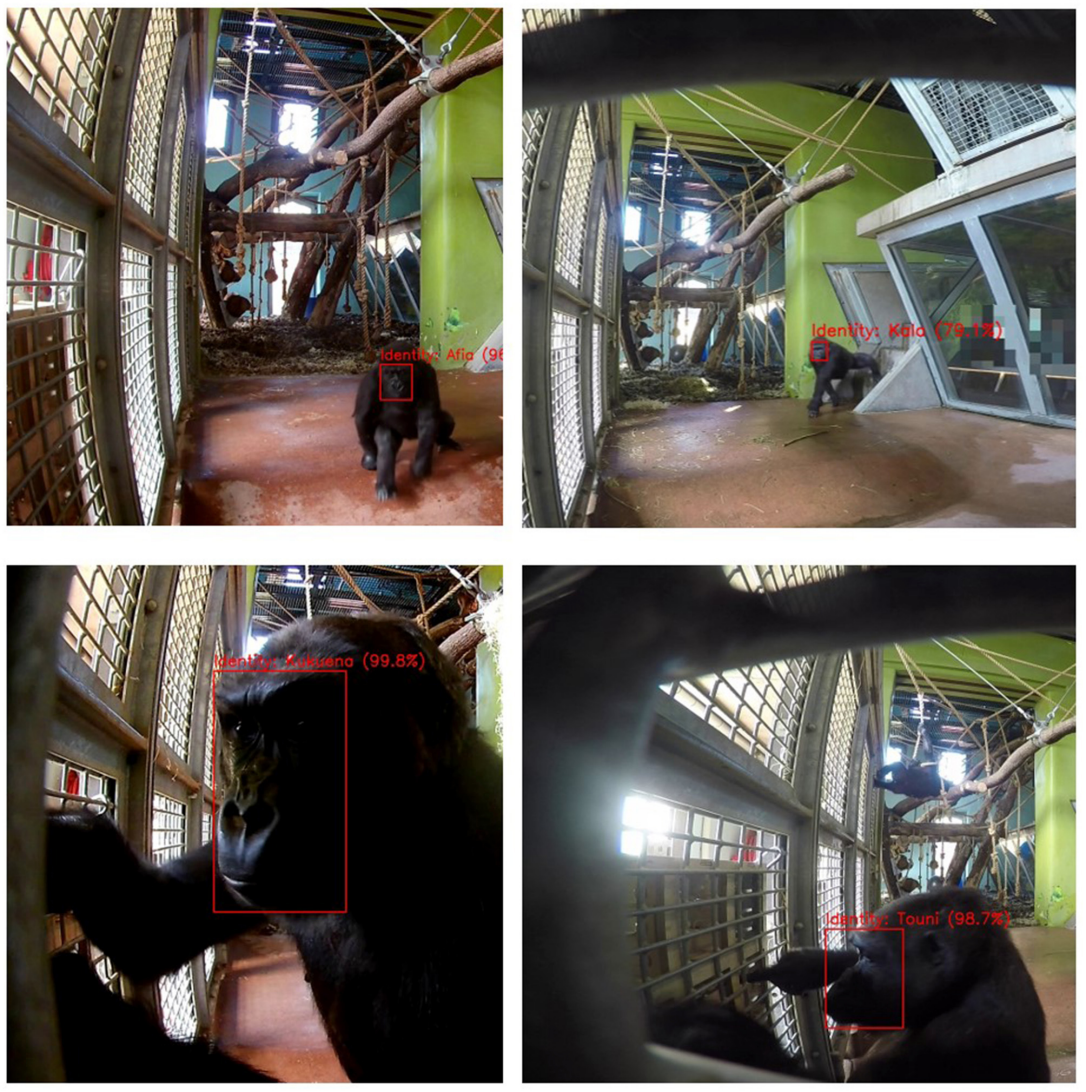
Single frame detection. The figure shows a collection of images of gorillas interacting with the Gorilla Game Lab device (on the left of each image) after processing by the facial recognition model. The red boxes represent facial localization predictions. The gorilla identities are also predicted by the model with the prediction confidence indicated as a percentage in brackets.

YOLOv3 was then trained on the dataset assembled previously. The specific training details are as follows. We used stochastic gradient descent (40) with momentum (41) of 0.9, batch normalization (42), learning rate decay (43) (an initial learning rate of 0.001 reduced by a factor of 10 at 80 and 90% of the total training iterations), batch size of 32 and an input resolution of 416 × 416 RGB pixels. The trained model forms the backbone of the facial recognition system by performing both localization and identification of gorilla faces. The output of the model is illustrated in Figure 5.

**Figure 6:**
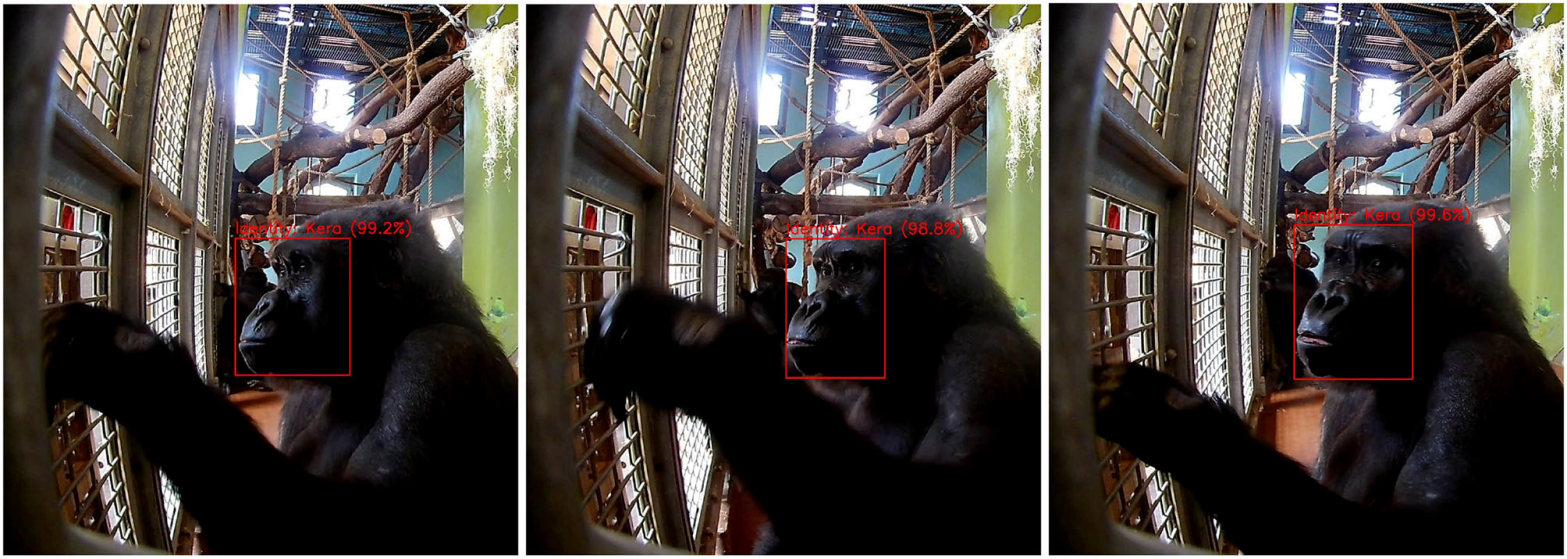
Tracklets and voting strategies. The figure shows a sequence of three frames (at time steps *t*_1_, *t*_2_ and *t*_3_) of a gorilla engaging with the Gorilla Game Lab device. The red boxes represent model predictions with the identity and confidence shown above the box, as in this figure. In this sequence of frames the true identity of the gorilla is Kera. The maximum voting strategy uses only the highest detection confidence (99.6%) at *t*_3_ to assign an identity across all time steps. The average voting strategy uses the highest average probability for *each* detected gorilla identity, where the mean is always calculated using the total number of time steps.

To further improve performance, we developed a multi-frame approach based on the single frame detector. The multi-frame approach functions by performing detection sequentially on multiple frames. As the performance of the detector is high (approx. 92% mAP) when applied to a single frame, the probability of generating incorrect predictions over several frames is low. Therefore, voting on the gorilla identity across frames yields improved performance. However, for this approach to be effective it is necessary to ensure that detections relate to the *same* individual across all frames. To do this we associate apes based on the similarity of their bounding boxes [i.e., intersection-over-union (IoU) of facial location] between frames, given gorillas will not move significantly from one frame to the next. Specifically, the trained YOLOv3 model was applied to individual frames of a sequence (i.e., video) where *X*_*t*_ denotes the frame at time step *t*. All detections in *X*_*t*_ and *X*_*t*+1_ were then input into a simple algorithm that associates apes *across* frames, leveraging temporal information to improve predictions. The algorithm associates apes which show the highest pairwise IoU, a measure of overlap between boxes, and exceeding an IoU threshold θ = 0.5. As the location of a gorilla is unlikely to change significantly between frames, association provides reasonable certainty that an individual gorilla in *X*_*t*_ and *X*_*t*+1_ is the same provided the IoU requirements are met. The resulting association chains represented tracklets (see Figure 6). Their length ranged from a single frame to 10 frames. For each tracklet, identity classification was evaluated *via* two methods: (1) maximum class probability; the highest single class probability is used as the identity of the gorilla for detections across all time steps, or (2) highest average class; the highest average probability is used as the identity of the gorilla for detections across all time steps.

## 3. Results

### 3.1. Behavioral Observation

The researchers found they could correctly identify a gorilla 100% of the time but it was not instantaneous; it took on average 5–10 s to identify individual gorillas from the inward facing video footage. On many occasions, a researcher began coding video footage of an “unknown” gorilla using the device, and waited until they could see a distinguishing characteristic (the face or a current body scar) to retrospectively confirm the gorilla's identity. Rarely, it was necessary to cross-check the footage from the outward facing GoPro camera to get a close view of a gorilla's face. It took on average 10–12 min to code 1 min of video footage. This refers to footage when a gorilla was using the device. When the device was not in use, there was nothing to code and the video was simply fast-forwarded on to the next bout of use.

### 3.2. Machine Learning

In this section, the results for the single-frame and multi-frame recognition models are reported, respectively. We use an evaluation protocol, the generation of train-test splits, that is standard within machine learning. In this protocol 20% of the manually annotated video footage is withheld. That is, the data is not seen by the model during training. This ensures that evaluation occurs on unseen data and that results are reported fairly. The test set comprises 1,105 images, as reported in Table 2. The facial location and gorilla identity in these images were labeled by a human with the assistance of the Primate Division at Bristol Zoo to ensure that all identities were assigned correctly. YOLOv3 is then applied to each of the images in the test set to generate facial location and individual identity predictions. These predictions were then evaluated against the human ground truth to measure model performance. We report performance using several benchmark evaluation metrics: individual average precision (AP), mean average precision (mAP), precision, and recall. Table 3 reports single frame classification performance of YOLOv3.

**Table 3:** Single frame YOLOv3 identification performance.

**Gorilla**	**AP (%)**	**Precision (%)**	**Recall (%)**
Afia	91.3	85.0	87.0
Ayana	74.9	84.0	68.0
Jock	98.5	98.0	92.0
Kala	92.7	95.0	89.0
Kera	97.2	96.0	92.0
Kukena	92.9	89.0	88.0
Touni	96.9	90.0	95.0
Mean	92.1 (± 8.0)	91.0 (± 5.5)	87.3 (± 9.9)

*The table depicts testing results for each of the individual gorillas*.

Table 4 reports multi-frame classification performance *via* precision, recall, and mAP for the test set, where the best performing single frame detector was used as the backbone of the system. The results reported utilize voting across a maximum tracklet size of 5, a stride of 1 and an IoU association threshold of 0.5. The multi-frame detector with maximum voting achieved the highest mAP, however, there was only a marginal difference between the maximum and average voting algorithms with less than 0.5% difference between all three of the reported evaluation metrics. Both multi-frame detection approaches outperformed the single frame detector across all metrics. The mAP improvements achieved by the average and maximum voting algorithms when compared with the single-frame detector were 5.2 and 5.4%, respectively.

**Table 4:** Multi-frame detector performance.

**Detection**	**mAP (%)**	**Precision (%)**	**Recall (%)**
Single	92.1 (± 8.0)	91.0 (± 5.5)	87.3 (± 9.9)
Average	97.3 (± 2.5)	95.1 (± 4.7)	91.1 (± 6.5)
Maximum	97.5 (± 2.2)	95.4 (± 2.7)	91.2 (± 7.9)

*Performance is shown for both average and maximum voting schemes. The performance of the single-frame detector is included for comparison. Note that the maximum voting scheme achieves the best performance across all evaluation metrics*.

We perform stratified five-fold cross-validation on both single-frame and multi-frame identification systems. We trained each fold for 24,000 iterations owing to time and computational restrictions. The three identification systems, single-frame and multi-frame identification with average and maximum voting schemes, achieved 89.91, 95.94, and 96.65% mAP, respectively.

## 4. Discussion

### 4.1. Traditional Observations

Traditional *post-hoc* coded video footage had a number of advantages and disadvantages in this study. The overarching advantage was that a researcher could record behaviors not directly classified as device usage but still crucial for the evaluation of cognitive enrichment; for example self-scratching and social play in close proximity (within one arm's length) of the device. These behaviors could not be detected through facial recognition. Behavioral observation is also “free”; it costs the researcher their time and some initial training to identify behaviors accurately but does not require any expensive technical equipment. While we used video cameras in this study, a researcher could have directly recorded gorilla behavior by eye if they wished.

Although the traditional approach was able to shed insights into how the device was being used, challenges remained. Firstly, the time required to analyse even a short duration of footage was substantial. Furthermore, the coding undertaken in this study could have been made more complex. For instance, to not just consider the direct interactions between gorillas and the enrichment device, but also to provide further depth of detail regarding the wider affect of the device upon the troop and their welfare. This raises questions regarding the feasibility of traditional approaches, particularly in under-resourced zoos without dedicated animal welfare teams or in circumstances where these teams must dedicate time to supporting multiple species.

There were also difficulties *in situ*. It was difficult for the researchers to use the video footage to code fine-scale data on how the gorillas were using the device. It was straightforward to record which of the 12 modules a gorilla was contacting and if a stick tool was present, but the precise motor skills being used and the number of finger/stick insertions was not always reliable, mainly due to the gorilla's posture and their body occluding most of their hand. Fluctuating light levels in the enclosure also contributed to shadowing and difficulty observing the modules. Another issue was potential human disturbance and gorilla responses to camera equipment. Even though the video camera was positioned in the public area of the enclosure (and was theoretically of no greater disturbance than normal visitor presence), the presence of a researcher/camera assistant in this area may have affected the gorilla's behavior. We were limited to using one small camera and tripod because the silverback male gorilla “Jock” had responded negatively to large camera rigs in the past. A small camera setup with no rigging or automation meant that the camera had to be manned constantly throughout 1 hr trials, increasing the time and effort of researchers.

### 4.2. Machine Learning

**Figure 7:**
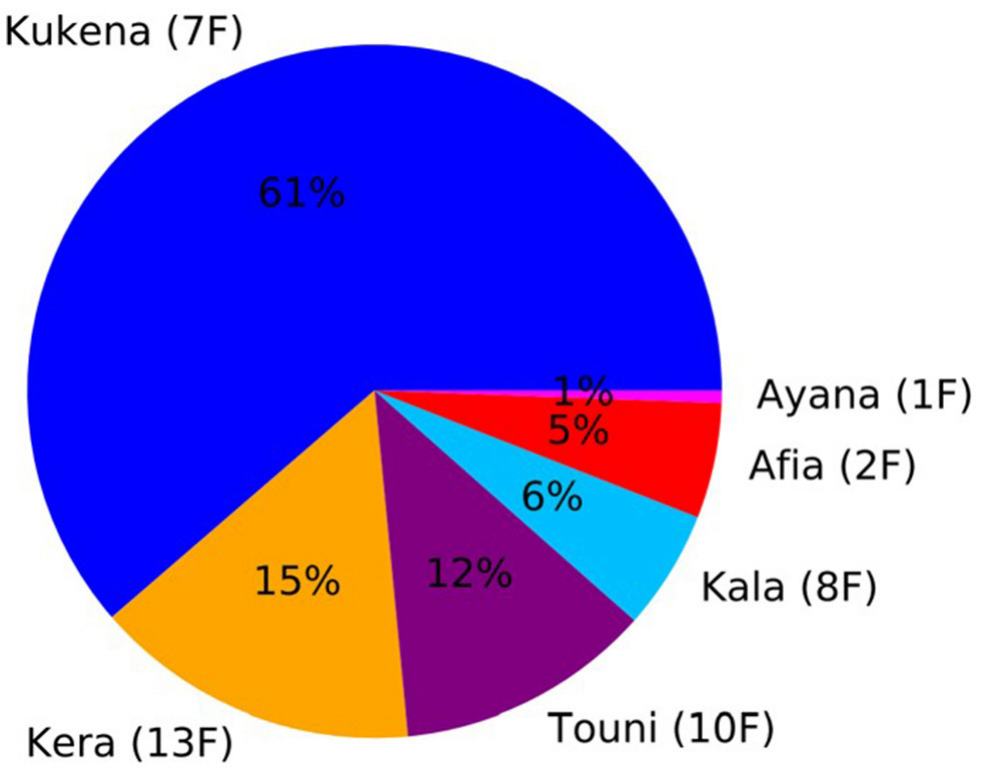
Usage Pie Chart. The figure shows the proportion of time spent by each gorilla engaging with the Gorilla Game Lab device over a 30-min segment. The age and sex of the gorilla is indicated in brackets (i.e., 1F indicates a 1 year old female). Kukena, 7 year old female and daughter of Jock, the male silverback, engages with the device the most. Ayana, 1 year old female, engages with the device the least.

**Figure 8:**
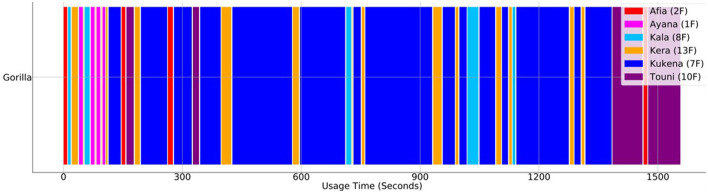
Order, Frequency & Duration of Use. The figure shows the order, duration and frequency each gorilla engaged with the Gorilla Game Lab device over the same 30-min segment as shown in Figure 7. The age and sex of the gorilla is indicated in brackets (i.e., 1F indicates a 1 year old female).

The experiments show that individual identification of gorillas can be performed robustly. The YOLOv3 object detector can be trained to perform simultaneous localization and classification of individual gorillas on single frames. Additionally, identification performance can be further improved by utilizing multiple frames. The single and multi-frame approach achieve 92.01 and 97% mAP, respectively. Therefore, the facial recognition application is capable of accurately identifying which, and to what extent, individual gorillas are engaging with the cognitive enrichment device; this allows usage frequency and duration to be monitored automatically. This can be done by applying the facial recognition system to new video footage and collecting the detections. By sampling the detections every second it is possible to automatically generate usage statistics. To illustrate this, our system was applied to a 30-min segment of unseen video footage collected at the zoo and detections were sampled every second, as described above. The detections were then processed using Python scripts to automatically generate usage statistic figures. Figure 7 shows the proportion of time each of the gorillas spent engaging with the device and Figure 8 indicates the order and frequency of use. The 30-min segment was manually verified by human observation.

**Figure 9:**
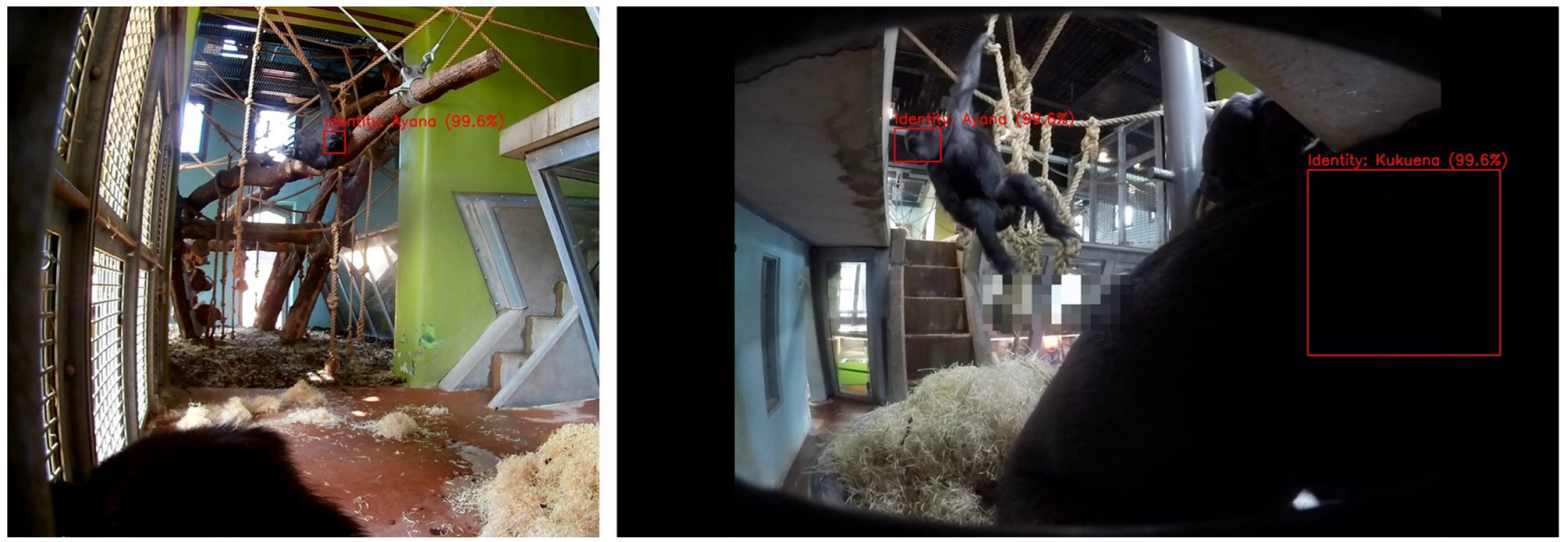
The figure shows cases where the detections do not represent true instances of interaction with the Gorilla Game Lab device. The image on the left shows a scenario where the gorilla interacting with the device is not detected due to crouching but the observing gorilla is. The image on the right shows simultaneous detection of the engaging gorilla and the observing juvenile gorilla.

There are, however, several aspects to consider with respects to the machine learning pipeline. The use of such a system introduces many interesting scenarios and where interpretation the of data is unclear. For example, only one gorilla can interact with the device at a given time. However, there are many scenarios where multiple gorillas are detected in frame. In most cases simply filtering scenarios where a gorilla is detected for a very short duration could remedy this scenario. However, there are instances where the gorilla interacting with the device is occluded and only the observing gorillas are detected. This could indicate incorrect periods of engagement by the observing gorilla. Similarly, juvenile gorillas frequently accompany more senior members of the troop for long duration's while they are engaging with the device. This can lead to multiple detections which occur simultaneously and do not represent true engagement. Examples of both these scenarios are shown in Figure 9.

Additionally, there are several key practical considerations to raise. Firstly, curating a custom dataset to train YOLOv3 is resource intensive, although usage data (frequency and duration) may be retrieved automatically thereafter. As a result of the erratic movements of the gorillas in their enclosure (i.e., rapid changes in poses, movement into spaces occluded by the mesh, some tampering with the camera-housing modules, etc.) it was necessary to manually view and select appropriate images. It was estimated that approximately 150 h were required to curate the dataset. Therefore, long-term projects, intended to span several months or years, would benefit from the described machine learning pipeline. However, short-term projects, lasting only several weeks, may be better suited to traditional behavioral observation. With that being said further experimentation shows that acceptable performance can be achieved with significantly less data (see Table 5), depending on the performance requirements of the study. Secondly, it does not automate collection of data such as strategy, module/s used or success. To collect this type of data, another method would need to be employed (such as observation, for example). Although this could be done alongside the described machine learning system, the time savings would not be as significant.

**Table 5:** Performance is shown for both average and maximum voting schemes.

	**Proportion of training data**			

	**10%**	**20%**	**40%**	**80%**
mAP	75.44 (± 12.2)	82.55 (± 9.94)	90.14 (± 6.66)	**92.02** (± 7.42)

*The performance of the single-frame detector is included for comparison. Note that the maximum voting scheme achieves the best performance across all evaluation metrics (as shown in bold)*.

Another important consideration is data management. Video footage recorded at high resolution (i.e., HD footage recorded at 30 FPS) has a large memory footprint. In this study, 30-min segments require approximately 5 GB of disk space. However, not all footage is necessarily of interest; there are large segments where no interaction is occurring. It is possible to utilize the machine learning system to identify segments containing no activity so they can be discarded, relieving some of the storage requirements. In any case, data storage should be considered ahead of time. In particular, studies which require all original footage to be retained should ensure a suitable storage solution is in place. In cases where it is not necessary to retain the original video footage, the meta-data produced by the machine learning system can be stored in light-weight storage formats such as JSON or XML. This storage format requires approximately 200 MB per 30-min segment. This could be further reduced by removing data points relating to periods of inactivity. Additionally, this data can be further condensed into summary statistics (i.e., total usage duration, usage order and usage duration for each gorilla interaction) and is all that is required to produce visualizations such as Figures 7, 8. Human data protection is also of utmost importance. In our study, even though we did not use any human data, we removed any small possibility of humans being identified in the background of footage using blurring software (Section 2.7.1). When working with animal image capture, researchers must make sure they have a procedure in place to deal with any inadvertant capture of human data.

Additionally, there are several key technical points to consider. Firstly, the dataset on which the model was trained and evaluated was gathered over a relatively short period of 6 weeks. Therefore, it is still possible that there is an overall lack of diversity in the data. For example, there will be large variations in lighting conditions, depending on the time of year, which may affect the performance of the system. It is uncertain therefore what, if any, the effect of this narrow data collection window has on the performance of the model and for future applications. Secondly, it is uncertain how changes in the appearance of gorillas over time will affect system performance. The appearance of the younger members of the troop will change relatively quickly as they grow and their facial features mature. It is possible that this could reduce the accuracy of the detector. The faces of the older members of the troop will also change as they age. Additionally, the behavioral patterns of the troop members may change over time and the class imbalances which exist in the dataset may no longer be reflective of the troop's dynamics. The device will need to be trialled over a long enough period for the effects of this to be evaluated.

Using the machine learning method, being able to identify the presence of specific individuals using the device helps to create a simple picture of the degree to which they are undertaking cognitive enrichment. This in itself could be used as a general marker of enrichment engagement for animal welfare staff within zoos. Additionally, the system is able to log the presence of gorilla interactions with enrichment across much larger time frames than would be possible with traditional observations alone. For example, while it may take 5–10 s to identify the gorillas in a single frame of footage manually, the same can be achieved by the facial recognition system in a fraction of this time. Additionally, the facial recognition system can operate continuously (and without downtime), irrespective of personnel, for as long as required. Yet, the machine learning method presently falls short of the behavioral observations with regards to the lack of qualitative understanding (i.e., the nature of gorilla interactions, approaches to problem solving, degree of success, or failure, etc.) generated about how the enrichment device is being used. With the variability of the challenging environment (e.g., camera occlusion, individual differences in movement, and approach to using the device), at present it is technically infeasible for our facial recognition to automate the analysis of gorilla behavior. Thus, the future direction of the project will consider a further ecosystem of technologies to understand this.

### 4.3. Future Directions

#### 4.3.1. Exploring Integrated Sensors

The facial recognition technology can robustly identify individuals and therefore be applied to determine which gorilla is engaging with the device and for how long. However, as stated, it does not provide any detail as to how the gorillas are engaging with the device. Thus, triangulating the facial recognition system with additional technology may produce richer insights. Sensor technology can detect physical phenomena, such as changes in light, temperature or pressure, and convert them into a machine readable signal. We speculate that sensor technology could be integrated into the device to automate tracking of gorilla device usage by monitoring each of the device's sub-modules individually. For example, by positioning a sensor in each of the sub-modules. Signals received from a sensor would indicate activity or interaction with a particular sub-module. Data on the individual usage of each sub-module would allow important cognitive and behavioral information to be deduced. For example, long periods of interaction with a specific module, relative to others, may indicate the difficulty level is too high.

We are conducting further research using infrared and piezo sensors and consider their efficacy for understanding enrichment device interactions in a small number of evaluations. In these evaluations, sensors were placed in each of the devices' sub-modules. The gorilla participants were presented with a maze of modules, of varying difficulty levels, which they were required to solve sequentially. It was necessary for each of the participants to extract a nut from the maze in the shortest possible time. At this stage, there is unfortunately no meaningful data that can be used to understand how the gorillas engaged with the device from the piezo sensors. This is a consequence of sound propagating through the device and obfuscating the results. For instance, a knock, scrape or manipulation in one module may show up across the adjacent module's sensors, without targeted interaction, leading to a lack of precision. On the other hand, preliminary results from the infrared sensor technology suggest that it is possible to gain an indication of how the gorillas are engaging with the device. The benefit of the infrared beam method for tracking gorilla device use was reliability; due to the size of the nuts being relatively regular, the infrared beam was very likely to register a nut falling between two modules. This allowed the timestamp of a triggering event to be logged and for the duration of maze use to be calculated. Subsequently, the average usage time per maze module could be determined.

#### 4.3.2. Toward a Zoo-Based Ecosystem of Technologies

As we build toward the triangulation of facial recognition and sensor technologies, we consider the wider implications this may have for the animal-centric design approaches. WAZA recommend that enrichment should be changed when appropriate to provide sufficient challenge and choice (44) and, thus, there is a push toward fine-tuning animal technologies toward the preferences of individual animals. In marrying facial recognition with efficacious sensor technologies, we aim to generate richer accounts of gorilla interactions with enrichment devices. This may allow us to develop more individually relevant experiences as a product of understanding the cognitive and affective consequences of our design decisions. We envisage that by combining the facial recognition and sensor technologies it would be possible to automatically determine windows of engagement for each gorilla and calculate the time spent on each maze during this window. With this information it is possible to build a personalized view of each gorilla and their individual enrichment needs that is based around their competencies and preferences. This would, in turn, allow more suitable configurations of maze modules to be presented to the gorillas and ultimately inform the design of new modules and future device iterations or modifications. A suite of evaluative technologies, as described above, would allow zoo keepers to infer optimal times to change enrichment and provide insight into the types of changes required.

While greater sensitivity to the enrichment needs of individual animals is a worthy endeavor, with the differences in resources between zoos, there is a need to make any ecosystem of technologies as accessible as possible for animal welfare staff. WAZA recommend building “staff skills, internal culture and commitment to enrichment strategies and activities” into daily management (44). Hence, in future directions of our design research, we must package our ecosystem of technologies to fit neatly within the daily animal welfare routines and culture of the zoo. One of the shortcomings of the methods outlined in this paper is their retrospective nature. Ideally, the gorilla identification and sensor data would be logged and displayed in real-time, allowing welfare staff to respond much more quickly to animal enrichment needs.

To make this accessible, we speculate that a virtual environment that can triangulate and present the facial recognition and sensor data could provide additional analytical automation. A second avenue of our ongoing research is the development of a dashboard application that could be used to allow the current and historical data to be visualized. Preferably a number of different “views” would be available to the user, allowing individual or troop-level statistics to be displayed. An application of this nature could provide keepers with the ability to assess (and possibly respond to) individual or collective enrichment needs with greater autonomy. Additionally, it may help to promote engagement with new enrichment strategies by allowing keepers to observe the effect of new maze module configurations or device modifications on the troop. Similarly, a greater understanding of how individuals in the troop engage with enrichment may lead to more effective and efficient deployment.

While, the sensors and wider triangulation of technologies may inspire new evaluative technologies for enrichment in zoos, they must undergo further development and rigorous evaluation. Data obtained from direct observation and sensors can yield very different results, so ultimately it is wise to use both if a research project can afford to do so, and until such a time that machine learning can fully replace live observations (45). Further thought is also required to ensure that our ecosystem of automated technologies can be deployed at scale and in zoos with varying resources. We recognize that this research project brings together an interdisciplinary team that would be hard to recreate and, thus, it is essential that our work builds toward accessible approaches. We look forward to presenting more detailed results of these avenues of research in future publications.

#### 4.3.3. Wider Implications of the Current Research

The system described in this project was developed specifically for gorillas. As described previously, methods for great ape facial recognition more generally have been borrowed from the human domain. They are assumed to be effective owing to similarities in their facial characteristics. Therefore, a similar system, dependent on facial recognition, could be implemented for other members of the great ape family, namely orangutans and chimpanzees. In particular, there is strong evidence to support the successful implementation of chimpanzee facial recognition systems (29, 31, 33). Similarly, there is evidence to suggest such a system may generalize and be effective for monitoring other primate species (46, 47). Additionally, such systems do not need to rely on facial recognition. Machine learning models can be trained, using the same protocol, to perform individual identification based on other features, such as full-body images (48, 49). This may be particularly useful where coat patterns, size and pose, rather than facial appearance, are more individually discriminative features. Furthermore, such a system is also suitable for use in other captive settings like sanctuaries, farms, and laboratories where granular individualized data may be of value in monitoring animal welfare. Lastly, machine learning could be used to generate data beyond individual identification; it also has the potential to generate detailed information on animal pose (22) and behavior (23, 50).

## Data Availability Statement

The datasets presented in this study can be found in online repositories. The names of the repository/repositories and accession number(s) can be found below: https://data.bris.ac.uk/data/dataset (and search BristolGorillas2020).

## Funding

This research was funded by the University of Bristol's Brigstow Institute, an initiative set up to support new collaborations between departments within the University of Bristol, and other academics working in Bristol and beyond. OB was supported by the UKRI Centre for Doctoral Training in Interactive Artificial Intelligence under grant EP/S022937/1.

## Author Contributions

OB and TB developed and validated the machine learning system and handled all machine learning data. SG, FC, KB, and PB developed and implemented the cognitive enrichment device. FC and KB developed the behavioral observation protocol, and behavioral data were collected by ER. SG structured the first draft of the manuscript. All authors contributed to the conception and design of the study, manuscript revision, read, and approved the submitted version.

## Conflict of Interest

The authors declare that the research was conducted in the absence of any commercial or financial relationships that could be construed as a potential conflict of interest.

## Publisher's Note

All claims expressed in this article are solely those of the authors and do not necessarily represent those of their affiliated organizations, or those of the publisher, the editors and the reviewers. Any product that may be evaluated in this article, or claim that may be made by its manufacturer, is not guaranteed or endorsed by the publisher.

## Footnotes

^1^ https://github.com/tzutalin/labelImg
